# The Predictive Role of Maternal Biological Markers and Inflammatory Scores NLR, PLR, MLR, SII, and SIRI for the Risk of Preterm Delivery

**DOI:** 10.3390/jcm11236982

**Published:** 2022-11-26

**Authors:** Ingrid Hrubaru, Andrei Motoc, Marius Liviu Moise, Bogdan Miutescu, Ioana Mihaela Citu, Raja Akshay Pingilati, Daniela-Eugenia Popescu, Catalin Dumitru, Florin Gorun, Flavius Olaru, Izabella Erdelean, Marius Forga, Nicoleta Nicolae, Cosmin Citu

**Affiliations:** 1 Department of Obstetrics and Gynecology, “Victor Babes” University of Medicine and Pharmacy Timisoara, Eftimie Murgu Square 2, 300041 Timisoara, Romania; 2 Doctoral School, “Victor Babes” University of Medicine and Pharmacy Timisoara, Eftimie Murgu Square 2, 300041 Timisoara, Romania; 3 Department of Anatomy and Embryology, “Victor Babes” University of Medicine and Pharmacy Timisoara, Eftimie Murgu Square 2, 300041 Timisoara, Romania; 4 Department of Radiology, “Premiere” Hospital—“Regina Maria”, Calea Aradului 113, 300643 Timisoara, Romania; 5 Department of Gastroenterology and Hepatology, “Premiere” Hospital—“Regina Maria”, Calea Aradului 113, 300643 Timisoara, Romania; 6 Department of Internal Medicine I, “Victor Babes” University of Medicine and Pharmacy Timisoara, Eftimie Murgu Square 2, 300041 Timisoara, Romania; 7 Malla Reddy Institute of Medical Sciences, Suraram Main Road 138, Hyderabad 500055, India; 8 Department of Neonatology, Premiere Hospital, Regina Maria Health Network, 300645 Timisoara, Romania

**Keywords:** pregnancy infections, premature birth, prematurity, inflammatory markers

## Abstract

In many countries, preterm birth, defined as birth before 37 completed weeks of gestation, is the primary cause of infant death and morbidity. An increasing body of research suggests that inflammation (both clinical and subclinical) plays a significant role in inducing preterm labor or developing pregnancy problems that lead to premature birth. Consequently, the purpose of this research was to determine the predictive value of the Neutrophil-Lymphocyte Ratio (NLR), derived Neutrophil-Lymphocyte Ratio (dNLR), Monocytes-to-Lymphocyte Ratio (MLR), Platelets-to-Lymphocyte Ratio (PLR), Systemic immune-inflammation index (SII), and systemic inflammatory response index (SIRI), for premature delivery. A retrospective study analyzed a total of 243 eligible pregnancies that resulted in a preterm birth during 2020 and 2021. A control group without a history of preterm birth was matched by age and trimester of laboratory analysis at a 1:1 ratio. Although the number of comorbidities was similar among study groups, the body-mass index estimated for the week of gestation was significantly higher among the patients from the prematurity group, as well as the prevalence of urinary tract infections and smoking. Laboratory data showed that patients with a preterm birth had significantly higher white blood cell count and monocytes, but significantly lower lymphocytes, platelets, and hemoglobin. The NLR, dNLR, PLR, and MLR scores showed to be significantly higher among patients from the prematurity group, but SII and SIRI were not significantly different between the study groups. It was observed that the AUC values of NLR, dNLR, PLR, and MLR were higher than 0.600, respectively NLR had the highest value among the tested scores (AUC = 0.694) and the highest sensitivity in this study (71%). The highest sensibility was achieved by dNLR, with 70%, and an AUC value of 0.655 (*p*-value = 0.022). PLR had the second-highest AUC value (0.682) and the best score in terms of sensitivity (70%) and sensibility (69%) (*p*-value = 0.015). Lastly, MLR had the lowest significant AUC score (0.607) and lowest sensitivity/sensibility. The significant cut-off values for the inflammatory scores were 9.0 for NLR, 9.8 for dNLR, 250 for PLR, and 4.07 for MLR. After evaluating the importance of these inflammatory scores, further clinical applications should be conducted to confirm the results and improve therapy and care to reduce the burden of premature deliveries.

## 1. Introduction

Preterm birth, defined as the onset of birth before 37 weeks of gestation, is a significant difficulty in obstetrics due to an estimated risk of 5 to 15 percent of all pregnancies resulting in preterm birth, which imposes a high cost on healthcare mostly owing to prolonged hospitalization, increased number of investigations performed, neonatal morbidity and death [1]. From the proportion of premature births of over 15 million worldwide, one million die due to severe prematurity, increased burden of complications, and lack of care in underdeveloped countries [2,3,4]. In addition to an increased risk of death, it has been shown that preterm infants suffer from a variety of complications and unfavorable outcomes with short and long-term implications [5,6].

Preterm birth might be medically induced owing to maternal or fetal indications, but about 70% occur spontaneously for no obvious reason [7,8]. The pregnancy period comes with a series of physiological adaptations that are well-known to be associated with different immune processes and elevated inflammatory status of the pregnant woman [9]. Since inflammation is believed to have a significant role in the initiation of labor in both preterm and term births, prior studies have focused on the variation in inflammatory markers and biological blood parameters to determine the link between serum markers of the pregnant woman and the risk of preterm birth [10,11]. It has been found that the number of macrophages increases in response to both term and preterm births, while neutrophils are more prevalent in the decidua of individuals with preterm births [12]. Therefore, it is plausible to postulate that an abnormally increased inflammatory status can trigger the moment of birth before the normal 37 weeks of gestation [13,14].

Predictive scores and forecasting tools have been widely implemented to aid in physicians’ decision-making processes by calculating the risk of a patient developing various outcomes [15,16,17,18]. For example, the neutrophil-to-lymphocyte ratio (NLR), platelet-to-lymphocyte ratio (PLR), and monocyte-to-lymphocyte ratio (MLR) are three biomarkers capable of predicting systemic inflammation that has recently gained interest because they are widely available markers that can be calculated from simple blood counts and show the prognostic significance for several diseases and outcomes [19,20]. In the area of obstetrics, however, research on the normal course of these conditions throughout pregnancy and their prognostic significance for pregnancy outcomes has been sparse. Only a few studies have evaluated, so far, the predictive usefulness of these biomarkers for birth outcomes, although with promising and accurate results [21,22]. It was described that PLR and NLR levels collected within one month previous to active labor were inversely linked with birthweight and gestational age. It was also found that elevated LMR levels during admission for suspected preterm labor were related to subsequent preterm birth in a high proportion of patients, therefore validating the predictive value of the score. However, these studies analyzed reduced sample sizes as well as a limited number of biological parameters as predictive scores. It hypothesized that calculating inflammatory scores during the second and third trimesters of pregnancy can identify good predictors. Based on these assumptions, the current study aimed to estimate the prognostic value of NLR, PLR, MLR, SII, and SIRI for preterm birth.

## 2. Materials and Methods

### 2.1. Study Ethics and Design

The research was conducted as an observational study at the Department of Obstetrics and Gynecology from the Victor Babes University of Medicine and Pharmacy in Timisoara, Romania. The study was designed as a retrospective cohort, with a data collection period spreading from the 1st of January 2020 until the 31st of December 2021. The study was approved by the Ethics Committee of the “Victor Babes” University of Medicine and Pharmacy and by the Ethics Committee of the Timisoara Municipal Hospital as the administrating institution of the patient database, being approved on the 19th of May 2022 with the code E-2814. 

### 2.2. Patient Inclusion and Study Groups

The current study aimed to analyze pregnant women with a history of preterm birth; therefore, we screened for all preterm births that occurred during the study period and included the adult patients that gave consent for data analysis with complete personal records. Incomplete digital or paper records, missing laboratory analysis, lack of consent, and underage patients were excluded from the study. Cases that presented with infections and inflammatory diseases were excluded from the study to avoid confounding factors. Other exclusion criteria comprised the use of anti-inflammatory and corticosteroid therapy during the sampling period, that might influence the biological parameters of the pregnant women and the computed inflammatory scores.

**Figure 1:**
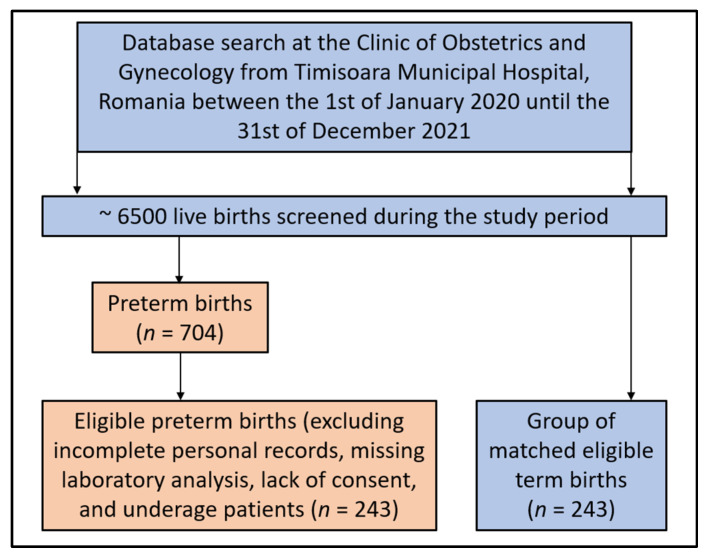
Flowchart of patients’ inclusion and exclusion criteria.

Using a convenience sampling method, it was determined for a 99% confidence level, a margin of error of 5%, and a population proportion of 10% premature births in the population of women of reproductive age, that a sample totaling 240 patients provides the minimum requirements for sufficient statistical power. Besides the reference group of pregnant women who gave birth prematurely, a control group without a history of preterm birth was matched by age and trimester of laboratory analysis at a 1:1 ratio and included in the current study for comparison, having the same inclusion criteria (Figure 1). 

### 2.3. Study Variables and Definitions

Premature birth was defined as a birth that occurred before 37 weeks of gestation, according to the World Health Organization guidelines [23]. The inflammatory scores were calculated as follows [24]: “NLR = absolute neutrophil count (ANC)/absolute lymphocyte count (ALC); derived Neutrophil-Lymphocyte Ratio (dNLR) = ANC/(WBC − ANC); MLR = absolute monocyte count divided/ALC; PLR = absolute platelet count (APC)/ALC; Systemic immune-inflammation index (SII) = (ANC × APC)/ALC; systemic inflammatory response index (SIRI) = (ANC × AMC)/ALC”. Laboratory values were randomly measured during the second trimester of pregnancy (13 to 26 weeks of gestation) or the third trimester of pregnancy (from 27 weeks of gestation until the moment of birth [25,26]. The study variables considered for statistical analysis comprised the following: demographic features and medical history (age, body mass index, number of pregnancies, number of births, number of comorbidities, urinary tract infections during pregnancy, history of pregnancy loss, history of abortion, SARS-CoV-2 infection during the pregnancy period, COVID-19 vaccination status, smoking status), and laboratory analysis (trimester of analysis, white blood cells, lymphocyte count, neutrophils, monocyte count, platelet count, hemoglobin, NLR, dNLR, PLR, MLR, SII, SIRI).

### 2.4. Statistical Analysis

The statistical analysis was performed with IBM SPSS v.27 (SPSS. Inc., Chicago, IL, USA), while the significance threshold was set for an alpha value of 0.05. The absolute and relative frequencies of categorical variables were computed and compared using the Chi-square and Fisher’s tests. The comparison of mean rank differences among nonparametric variables was performed with the Mann–Whitney U-test. Parametric continuous variables that followed a normal distribution were compared by mean and standard deviation with the Student’s *t*-test. A Kaplan–Meier curve was plotted for the probability of prematurity, while the Cox regression identified the hazard ratio for prematurity. A multiple logistic regression analysis was performed to calculate the risk (OR) for prematurity using the NLR, PLR, MLR, SII, and SIRI as predictors, adjusted by variables with significant differences between the two study groups. Using the receiver operating characteristic (ROC) curve approach, the prediction performance of the risk of preterm birth was evaluated by calculating the area under the curve (AUC) and its associated significance value. Using Youden’s index, the appropriate cut-off values for inflammatory indices were calculated.

## 3. Results

### 3.1. Background Analysis

The study comprised 486 patients analyzed for changes in biological parameters during the pregnancy period in order to determine the predictive role of various inflammatory scores computed from the basic serum biomarkers. Patients were split into two equally matched study groups, a reference group of 243 pregnant women who gave birth prematurely and 243 pregnant women who gave birth at full term. The background analysis presented in Table 1 showed no differences in the mean age of participants, as they were matched by age, with 29.6 years in the prematurity group vs. 29.9 years in the full-term birth group. However, the body-mass index estimated for the week of gestation was significantly higher among the patients from the prematurity group (26.2 kg/m^2^ vs. 22.4 kg/m^2^, *p*-value < 0.001). The number of comorbidities did not differ significantly between the study groups, although pregnant women in the prematurity group had a significantly higher proportion of urinary tract infections during the current pregnancy that resulted in a premature birth (18.1% vs. 11.5%, *p*-value = 0.041). Additionally, since the study was conducted during the COVID-19 pandemic, it was observed that a higher proportion of patients in the prematurity group had COVID-19 during the pregnancy period (7.0% vs. 2.5%, *p*-value = 0.021). Lastly, from the background analysis, there were significantly more smoking patients in the prematurity group as compared to the full-term group (*p*-value = 0.006). 

### 3.2. Laboratory Analysis

Table 2 describes the laboratory analysis of pregnant women included in the study and the calculated inflammatory scores. A total of 209 patients had their blood samples taken during the second trimester of pregnancy, while the other 277 were measured during the third trimester, with no significant changes between the two study groups. Among biological markers, it was observed that the white blood cell count, lymphocyte count, monocyte count, and the number of platelets had significantly different average values. Additionally, anemia was significantly more common among patients with premature birth, with a hemoglobin level of 11.72 g/dL, compared to 12.99 g/dL (*p*-value < 0.001). Regarding the inflammatory scores calculated for each study group, it was observed that NLR (13.75 vs. 9.06, *p*-value < 0.001), dNLR (6.92 vs. 5.11, *p*-value < 0.001), PLR (286.2 vs. 237.0, *p*-value = 0.007), and MLR (0.86 vs. 0.79, *p*-value = 0.005) scores were significantly higher among those who had a preterm birth.

### 3.3. ROC and AUC Analysis

**Figure 2:**
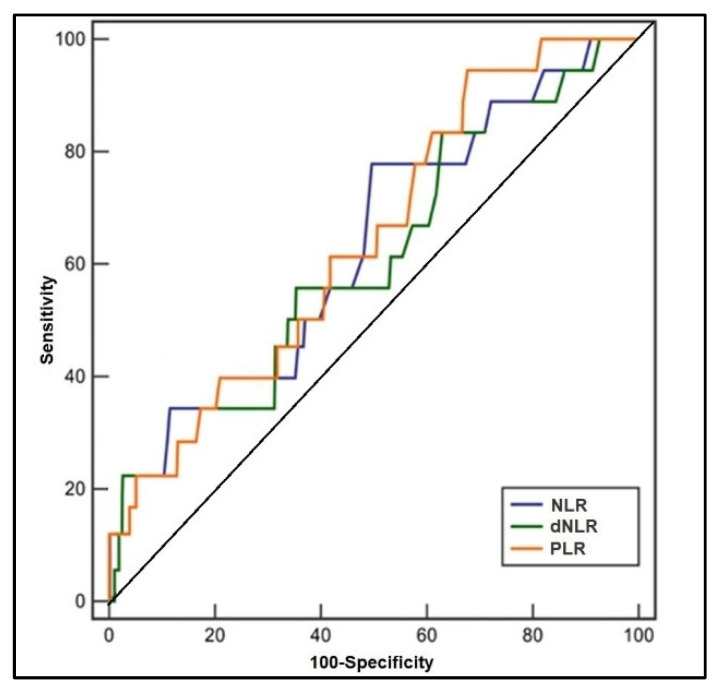
Receiver operating characteristic plots of NLR, dNLR, and PLR in predicting premature birth.

**Figure 3:**
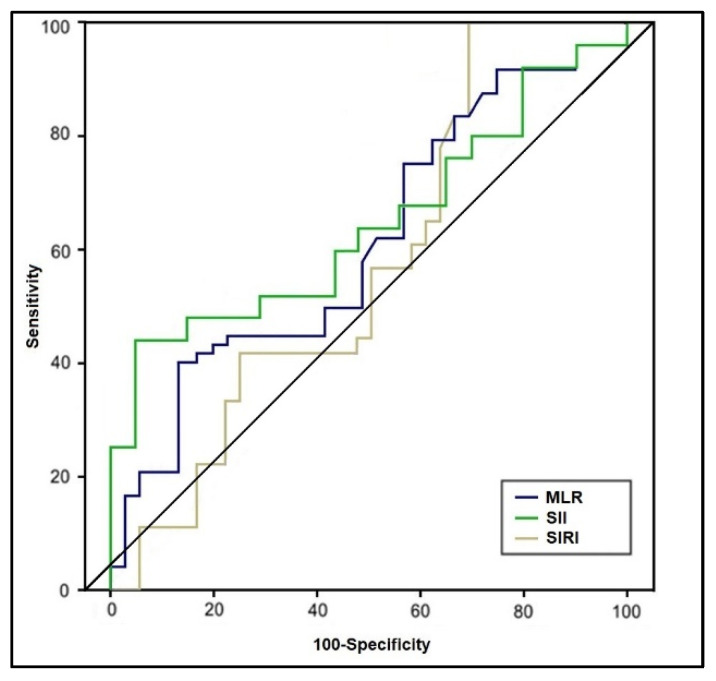
Receiver operating characteristic plots of MLR, SII, and SIRI in predicting premature birth.

The ROC analysis and the computed areas under the curve are presented in Table 3, respectively Figure 2 and Figure 3, representing the predictive role of computed inflammatory markers in premature birth. It was observed that the AUC values of NLR, dNLR, PLR, and MLR were higher than 0.600, respectively NLR had the highest value among the tested scores (AUC = 0.694, *p*-value = 0.009), with the highest sensitivity in this study (71%). The highest sensibility was achieved by dNLR, with 70%, and an AUC value of 0.655 (*p*-value = 0.022). PLR had the second-highest AUC value (0.682) and the best score in terms of sensitivity (70%) and sensibility (69%) (*p*-value = 0.015). Lastly, MLR had the lowest significant AUC score (0.607) and lowest sensitivity/sensibility values among the statistically significant scores (*p*-value = 0.048). SII and SIRI scores had computed AUC values below 0.600 without statistical significance.

### 3.4. Risk Analysis

**Figure 4:**
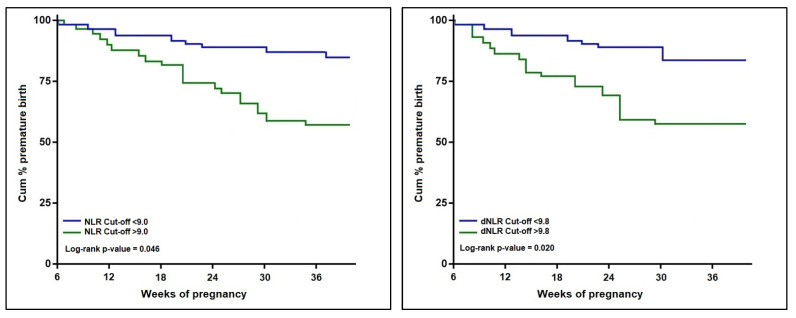
Kaplan–Meier probability analysis of premature birth by NLR and dNLR scores.

**Figure 5:**
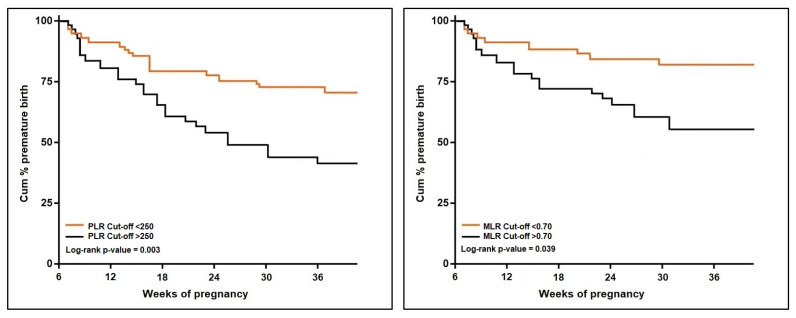
Kaplan–Meier probability analysis of premature birth by PLR and MLR scores.

The univariate Cox regression analysis described in Table 4 and Figure 4 and Figure 5 calculated a hazard ratio for premature pregnancy of 3.61 (*p*-value < 0.001) for an NLR score over 9.0 (log-rank *p*-value = 0.046). The risk was 3.13 times higher when a dNLR score surpassed the cut-offcut-off value of 9.8 (log-rank *p*-value = 0.020). The PLR risk was the highest among the calculated scores, with an HR of 4.07 (*p*-value < 0.001), over the threshold of 250 (log-rank *p*-value = 0.003). Lastly, an MLR score higher than 0.70 posed a 1.96 times higher risk for premature pregnancy (log-rank *p*-value = 0.039). The SII and SIRI scores were eliminated from the probability analysis since they did not show significant results.

## 4. Discussion

### 4.1. Important Findings

The current study identified that the NLR, dNLR, PLR, and MLR scores showed to be significantly higher among patients who gave birth prematurely, but SII and SIRI were not significantly different between the study groups. NLR had the highest value among the tested scores and the highest sensitivity in this study (71%). Similar to our results, several investigations assessed the inflammatory scores for premature membrane rupture, a prelude to preterm delivery. NLR and PLR were discovered to be considerably greater in the PPROM group compared to the control group; nevertheless, sepsis was more prevalent in the PPROM group. PLR levels were substantially associated with an increased risk of preterm premature rupture of membranes, while the cut-off value in our analysis was 250, whereas, in the other study, it was 117 [27].

NLR is involved in inflammatory processes since the differentiation of leukocyte subtypes is an immunological response that occurs in settings characterized by systemic inflammation. As a consequence of this, the NLR has the propensity to change in a variety of systemic inflammatory disorders. Numerous studies have shown that an elevated NLR has both a prognostic and a predictive value in malignancies [28,29]. Additionally, NLR was shown to be considerably changed in a variety of pregnancy-related diseases, as some authors found that preeclampsia was significantly associated with elevated NLR levels [30]. Under similar circumstances, NLR levels were discovered to be changed in pregnant patients with gestational diabetes, intrahepatic cholestasis, hyperemesis gravidarum, and acute appendicitis of pregnancy [31,32]. In another research, the NLR was shown to be significantly greater in the preterm group than in the controls, concluding that NLR was a valuable and accurate marker for PPROM prediction [33]. 

Current research has emphasized the efficacy of the NLR in predicting preterm birth in conjunction with other events occurring during pregnancy. Regarding other pathologies, one study found that a model including cervical length and NLR had a greater diagnostic and prognostic value for preterm birth than cervical length alone or other systemic inflammatory indicators such as CRP and leukocyte levels [34]. Another research showed that increased NLR levels are related to preterm births and neonates with lower birth weight [35]. The authors predicted that a hyperinflammatory condition in the mother, as indicated by a high NLR, might contribute to fetal development disturbances, resulting in low birth weight and early delivery onset. However, a wide populational study by Morisaki et al. that studied more than 70 thousand pregnancies observed that although NLR and PLR could be regarded as predictor scores for preterm labor, the prediction value was significant only in association with ischemic placental disease [21]. Therefore, when reporting these scores such as in our study, the physician should always consider the possibility of a preexistent factor. 

Although our study did not specifically create a prediction model as an association of multiple inflammatory markers that correlated with the risk of preterm labor, a recent research described that the correlation between NLR, hemoglobin, and platelet distribution width (PDW) marker successfully forecasts premature delivery [19]. The score correlated significantly with preterm birth at a cutoff value of 0.25, with sensitivity and specificity of 88.6% and 40.5% and negative and positive predictive values of 97.9% and 10.0%, respectively. This association of serum parameters may complement other indicators in predicting PTB around 10 weeks beforehand. This marker combination has a very high negative predictive value for preterm birth. In people with a normal composite marker result, further PTB screening tests may be omitted unless clinical suspicion is severe. Prospective investigations are required to determine whether there are placental pathologic alterations associated with maternal vascular hypoperfusion, which may explain the connection between NLR and premature birth.

PLR is a marker that started to be used in clinical practice since it has been shown to accurately predict thrombotic events and inflammatory illnesses. A strong relationship between higher PLR and severe outcomes in cardiovascular illnesses and lower survival in malignancies such as endometrial cancer was revealed by a number of studies that were conducted in the past [36,37]. PLR was examined in gestational diabetes, acute pancreatitis, preeclampsia, and PPROM in women who were pregnant, although it did not demonstrate any significant differences between oligohydramnios and normal amniotic fluid index groups [38]. Regarding the latency period, another research was conceived of with the intention of investigating the connection that exists between PLR, PPROM, and preterm birth, showing that there was no significant difference in PLR across latency intervals of less than 72 h and more than 72 h. However, in our research, PLR showed a significant prognostic value with good accuracy for preterm birth.

Other recent studies attempted to determine the role of inflammatory scores such as MLR and SIRI, but for different outcomes, such as mortality in COVID-19 patients. It was observed that MLR and SIRI scores above the threshold of 0.69, respectively 2.2 were predicting with significant accuracy the mortality in hospitalized patients with severe SARS-CoV-2 infection [24,39]. Moreover, another study found that Elevated SII scores were associated with a substantially worse P/F ratio and chest CT severity score, suggesting that SII may represent the pulmonary and respiratory injury occurring in COVID-19 patients rather than a general impairment of their clinical conditions due to comorbidities [40].

Previous studies have thoroughly documented the role of comorbidities and infections during the pregnancy period as significant risk factors for giving birth prematurely [40,41,42]. Although patients in our study were matched by the number of comorbidities to eliminate the bias risk, those who gave birth prematurely were more likely to be overweight. It was previously demonstrated that obesity plays a significant role in developing comorbidities associated with an elevated inflammatory status [43]. Another significant factor that might contribute to a premature birth is the SARS-CoV-2 infection that was recently described in several studies suggesting that pregnant women with COVID-19 during the third trimester of pregnancy are statistically significantly more likely to give birth prematurely [44,45]. 

Although several studies have reported that SARS-CoV-2 infection during pregnancy is associated with a dangerously higher inflammatory status calculated by the NLR and PLR scores as significant predictors for preterm birth [46,47], some wider studies described the opposite. The multicentric study by Piekos et. al, as well as a retrospective study by Guelersen et al. found that after first or second trimester SARS-CoV-2 infection, pregnant women would benefit from additional surveillance and improved prenatal care, independent of the degree of acute SARS-CoV-2 infection. Early preterm women are substantially less likely to develop PTB after hospitalization for SARS-CoV-2 infection than their late preterm counterparts. The bulk of PTB were suggested and not spontaneously induced [48,49]. 

Even though inflammatory scores such as NLR and PLR have a significant prediction value for premature birth in our research, other studies found important limitations of these scores, but for different outcomes, such as mortality. For example, the poor accuracy of PLR in determining in-hospital mortality during a short period of follow-up may be due to the kinetic fluctuation of the platelet count, implying possibly the influence of the drop in lymphocyte count, which is an independent predictor of death, and time might be a confounding variable, it was hypothesized that PLR’s prediction improved the closer it was to the given result. Nevertheless, in forecasting mortality, PLR is shown to be less reliable than NLR [50,51].

### 4.2. Study Limitations and Future Perspectives

Starting with the retrospective nature of the investigation, this article contains various disadvantages. Since data were collected from a single clinic, the group analyzed may be more homogeneous and less generalizable to the larger population. Another limiting factor of the retrospective design was that blood samples could not be drawn at specific moments to exclude some confounding factors, as it could be done in a prospective study. In addition, we could not rule out the influence of certain therapies on the observed inflammatory marker outcomes. There are still biological indicators that have not been fully investigated, but they could be able to better predict the fate of the pregnancy and enable better care. Since anemia was more common in the studied patients who gave prematurely in this study, future research should investigate predictive scores based on hemoglobin levels, red blood cell count, and other red blood cell parameters.

## 5. Conclusions

The inflammatory scores NLR, dNLR, PLR, and MLR measured throughout the second and third trimesters of pregnancy exhibited a high predictive value for preterm delivery. Future clinical research should study techniques to diminish the impacts associated with high levels of these indices in order to enhance therapies and management in order to lessen the burden of preterm. 

## Figures and Tables

**Table 1:** Background characteristics of the patients analyzed in the study.

Variables *	Prematurity Group (*n* = 243)	No Prematurity Group (*n* = 243)	Significance
General characteristics			
Age (years), mean ± SD	29.6 ± 4.9	29.9 ± 5.0	0.504
BMI (kg/m^2^), mean ± SD	26.2 ± 3.3	22.4 ± 3.1	<0.001
Previous pregnancies			0.892
1	152 (61.3%)	157 (61.7%)	
2	62 (23.4%)	59 (25.9%)	
≥3	29 (15.3%)	27 (12.3%)	
Number of births			0.099
1	187 (77.0%)	171 (70.4%)	
≥2	56 (23.0%)	72 (29.6%)	
Comorbidities **			0.274
0	176 (72.4%)	189 (77.8%)	
1	54 (22.2%)	40 (16.5%)	
≥2	13 (5.3%)	14 (5.8%)	
Obstetrical characteristics			
Week of birth, mean ± SD	35.9 ± 4.7	37.7 ± 5.1	<0.001
PPROM	18 (7.4%)	4 (1.6%)	0.002
Abnormal placental implantation	24 (9.9%)	18 (7.4%)	0.332
Cesarean delivery	36 (14.8%)	41 (21.0%)	0.075
UTIs during pregnancy	44 (18.1%)	28 (11.5%)	0.041
History of pregnancy loss	4 (5.8%)	9 (3.7%)	0.285
History of induced abortion	11 (4.5%)	7 (2.9%)	0.336
COVID-19 during pregnancy	17 (7.0%)	6 (2.5%)	0.018
COVID-19 vaccination status	31 (12.8%)	44 (18.1%)	0.102
Smoking status	24 (9.9%)	9 (3.7%)	0.006

* Data is presented as *n* (%) unless specified differently; ** Including diabetes mellitus, asthma and other respiratory disease, coagulation disorders, high blood pressure and cardiovascular disease, endocrine disorders, recurrent urinary tract infections, chronic viral infections, and depression. BMI—Body Mass Index; UTI—Urinary Tract Infections.

**Table 2:** Laboratory analysis of pregnant women included in the study.

Variables *	Prematurity Group (*n* = 243)	No Prematurity Group (*n* = 243)	Significance
Trimester of analysis			0.783
2nd trimester	103 (42.4%)	106 (43.6%)	
3rd trimester	140 (57.6%)	137 (56.4%)	
Serum parameters			
WBC (×10^9^/L)	9.22 ± 5.70	8.94 ± 5.21	0.048
Lymphocytes (×10^9^/L)	0.76 ± 0.48	1.05 ± 0.89	<0.001
Neutrophils (×10^9^/L)	8.10 ± 5.13	7.24 ± 4.97	0.061
Monocytes (×10^9^/L)	0.56 ± 0.17	0.52 ± 0.19	0.014
PLT (×10^9^/L)	210.8 ± 72.3	232.1 ± 79.6	0.002
Hb (g/dL)	11.72 ± 1.54	12.99 ± 1.60	<0.001
Inflammatory scores			
NLR	13.75 ± 9.13	9.06 ± 7.17	<0.001
dNLR	6.92 ± 3.17	5.11 ± 3.09	<0.001
PLR	286.2 ± 195.4	237.0 ± 203.8	0.007
MLR	0.86 ± 0.33	0.79 ± 0.21	0.005
SII	2351 ± 1044	2185 ± 1142	0.095
SIRI	6.94 ± 4.86	6.12 ± 4.91	0.064

* Data is presented as *n*(%) unless specified differently; PLT—Platelets; WBC—White Blood Cells; Hb—Hemoglobin; NLR—Neutrophil-Lymphocyte Ratio; dNLR—derived Neutrophil-Lymphocyte Ratio; PLR—Platelets-Lymphocyte Ratio; MLR—Monocytes-Lymphocyte Ratio; SII—Systemic immune-inflammation index; SIRI—systemic inflammatory response index.

**Table 3:** ROC plot of the optimal inflammatory scores.

Inflammatory Scores	AUC	95% CI		SE	Sensitivity	Specificity	Significance
		Lower Bound	Upper Bound				
NLR dNLR	0.694 0.655	0.561 0.538	0.843	0.078	71%	66%	0.009
			0.822	0.074	65%	70%	0.022
PLR	0.682	0.556	0.857	0.081	70%	69%	0.015
MLR	0.607	0.462	0.705	0.093	66%	63%	0.048
SII	0.580	0.494	0.736	0.125	52%	65%	0.113
SIRI	0.496	0.317	0.692	0.183	48%	69%	0.157

ROC—Receiver Operating Characteristic; AUC—Area Under Curve; CI—Confidence Interval; SE—Standard Error; NLR—Neutrophil-Lymphocyte Ratio; dNLR—derived Neutrophil-Lymphocyte Ratio; PLR—Platelets-Lymphocyte Ratio; MLR—Monocytes-Lymphocyte Ratio.

**Table 4:** Regression analysis for risk of premature birth in SARS-CoV-2 infected pregnant women.

	Risk	(95% CI)	Significance
Hazard Ratio			
NLR	3.61	1.94–6.15	<0.001
dNLR	3.13	1.82–5.34	<0.001
PLR	4.07	1.25–7.84	<0.001
MLR	1.96	1.44–3.78	0.002
SII	1.50	0.94–1.45	0.134
SIRI	1.24	0.92–1.97	0.090
Adjusted Odds Ratio *			
NLR	4.23	1.81–7.36	<0.001
dNLR	3.09	1.72–5.94	<0.001
PLR	5.65	2.30–8.05	<0.001
MLR	2.17	1.39–2.51	0.046
SII	1.58	0.99–1.93	0.217
SIRI	1.66	0.89–1.87	0.195

CI—Confidence Interval; NLR—Neutrophil-Lymphocyte Ratio; dNLR—derived Neutrophil-Lymphocyte Ratio; PLR—Platelets-Lymphocyte Ratio; MLR—Monocytes-Lymphocyte Ratio; * Adjusted for BMI, smoking and COVID-19 infection status.

## Data Availability

The data presented in this study are available on request from the corresponding author.
